# Non-responders to topical Imiquimod followed by vaccination therapy in VIN patients may be due to the level of IL10

**DOI:** 10.1038/sj.bjc.6605824

**Published:** 2010-07-27

**Authors:** X S Liu

**Affiliations:** 1 Graduate School of Medicine, University of Wollongong, NSW, Australia; 2 Illawarra Health and Medical Research Institute, University of Wollongong, NSW, Australia

**Sir,**

Vulval intraepithelial neoplasia (VIN) is resulted from persistent human papillomavirus (HPV) infection, especially infection of HPV type 16 (HPV16) (Trimble and Frazer, 2009). Several studies have shown the effectiveness of topical administration of Imiquimod, a Toll-like receptor 7 agonist, for the treatment of VIN (Davis *et al*, 2000; Diaz-Arrastia *et al*, 2001). Recently, these results are confirmed by a randomized, placebo-controlled clinical study (van Seters *et al*, 2008). The HPV16-related Th1 responses, especially local Th1 responses, are critical for the favoured clinical outcome after Imiquimod treatment (van Poelgeest *et al*, 2005; van Seters *et al*, 2008).

Daayana *et al* (2010) further showed in a paper published in *British Journal of Cancer* that Imiquimod topical application followed by HPV therapeutic vaccine led to the complete regression of VIN in 63% of patients recruited in a Phase II clinical trial at 52 weeks after the treatment. There was significantly increased local infiltration of CD8 and CD4 T cells in responders; whereas non-responders showed an increased density of T regulatory cells in the lesions (Daayana *et al*, 2010). The authors speculated that genetic predisposition involving immune and other parameters may be responsible for the differences between responders and non-responders. Similar clinical outcome was observed in VIN patients treated with Imiquimod topical application followed by photodynamic therapy from the same group (Winters *et al*, 2008), although the underlying mechanisms of therapeutic vaccination and photodynamic treatment after Imiquimod application may be different. Interestingly, the non-responders also showed a significantly higher level of T regulatory cells in the lesions after Imiquimod treatment (Winters *et al*, 2008).

Activation of TLRs not only induces inflammatory cytokines, such as TNF*α* and IFN*γ*, but also promotes the secretion of IL10, an inhibitory cytokine that damps immune responses (Saraiva and O’Garra, 2010). Topical application of Imiquimod to a transgenic mice bearing HER-2/neu+ breast cancer induces high level of IL10 (Lu *et al*, 2010), which promotes tumour progress after the treatment is ended. The IL10 is secreted by a population of CD4+ T cells also secreting IFN*γ*, and these cells are most likely responsible for the ineffectiveness of Imiquimod therapy. The IL10 secreting T regulatory cells can be amplified by the subsequent vaccination (Zhou *et al*, 2006). Blocking IL10 increases cytotoxic T cells responses activated through TLRs and enhances the antitumour effect of Imiquimod by significantly prolonging survival in treated mice (Liu *et al*, 2006; Lu *et al*, 2010).

IL10 polymorphism influences IL12 secretion by dendritic cells in response to the TLRs stimulation (Peng *et al*, 2006), thus control the level of Th1 responses, which is critical for control tumour growth. It is likely that in non-responder patients, the CD4+ T cells secrete more IL10 in response to Imiquimod application, prevent the generation of CD4 and CD8 effector T cells that are able to migrate to the tumour site to kill tumour cells. It is worth investigating the level of IL10, and IL10 polymorphism after Imiquimod application in responders and non-responders; and whether blocking IL10 signalling at the time of Imiquimod application further improves the efficacy of the treatment.
